# Vanishing lung syndrome in a patient with HIV infection and recurrent pneumothorax

**DOI:** 10.11604/pamj.2017.27.141.13046

**Published:** 2017-06-28

**Authors:** Saqib Saeed, Sanjiv Gray

**Affiliations:** 1 Department of Surgery, Harlem Hospital Columbia University Medical Center, New York, NY, USA

**Keywords:** Pneumothorax, HIV, emphysema

## Image in medicine

Vanishing lung syndrome (VLS) is a rare chronic, progressive clinical entity seen in smokers characterized by giant emphysematous bullae mainly in the upper lobes. Extensive paraseptal emphysema coalesces to form giant bullae, compressing the normal lung parenchyma. The criteria for diagnosis based on radiography was defined by Roberts et al in 1987 and includes the presence of giant bulla in one or both upper lobes, occupying at least one third of the hemithorax and compressing the surrounding normal lung parenchyma. HIV infection can worsen the disease process. We report a 56 year-old HIV positive male with a smoking history of 30 pack-years smoking history who presented to the Emergency room with chronic chest pain, dyspnea, and cough for 2-3 weeks duration. He had a history of COPD and had multiple prior admissions for recurrent pneumothoraces. Chest radiography (A) showed hyperlucent and hyperinflated lung with bilateral pleural thickening and bilateral severe bullous emphysematous changes which was suggestive of vanishing lung syndrome. Computed tomography of the chest confirmed these findings (B). Chest tube was placed on the left side for treatment of the pneumothorax. The patient had refused lung reduction surgery in the past. He was counseled on smoking cessation and was discharged home with a Heimlich valve catheter. The main complication of VLS is pneumothorax. Lung reduction surgery such as bullectomy results in improvements in dyspnea, gas exchange, and exercise tolerance. Clinical should be aware of the condition and early referral for lung reduction surgery is required. Smoking cessation is essential.

**Figure 1:**
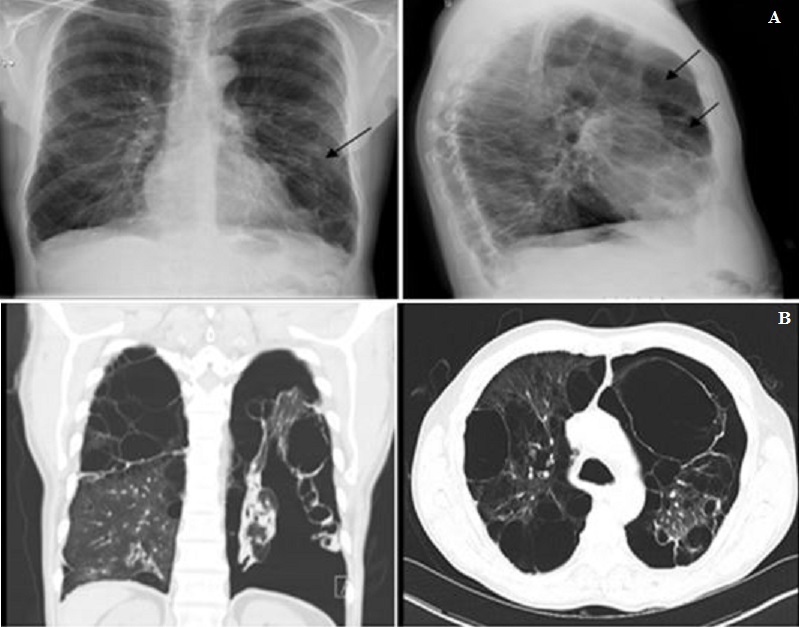
(A) PA and lateral views of the chest X-ray showing severe emphysematous changes; (B) axial and coronal views of the chest CT showing extensive bilateral bullous lung disease with collapse of surrounding parenchyma

